# Developing pangenomes for large and complex plant genomes and their representation formats

**DOI:** 10.1016/j.jare.2025.01.052

**Published:** 2025-01-31

**Authors:** Pradeep Ruperao, Parimalan Rangan, Trushar Shah, Vinay Sharma, Abhishek Rathore, Sean Mayes, Manish K. Pandey

**Affiliations:** a Center of Excellence in Genomics and Systems Biology (CEGSB) and Center for Pre-Breeding Research (CPBR), International Crops Research Institute for the Semi-Arid Tropics (ICRISAT), Hyderabad, India; b ICAR-National Bureau of Plant Genetic Resources (NBPGR), New Delhi, India; c Queensland Alliance for Agriculture and Food Innovation, The University of Queensland, St Lucia, Australia; d International Institute of Tropical Agriculture (IITA), Nairobi, Kenya; e International Maize and Wheat Improvement Center (CIMMYT), Nairobi, Kenya

**Keywords:** Pangenome, Graph format, PHG, Haplotype graph, Genome viewer, Graph viewer

## Abstract

**Background:**

The development of pangenomes has revolutionized genomic studies by capturing the complete genetic diversity within a species. Pangenome assembly integrates data from multiple individuals to construct a comprehensive genomic landscape, revealing both core and accessory genomic elements. This approach enables the identification of novel genes, structural variations, and gene presence-absence variations, providing insights into species evolution, adaptation, and trait variation. Representing pangenomes requires innovative visualization formats that effectively convey the complex genomic structures and variations.

**Aim:**

This review delves into contemporary methodologies and recent advancements in constructing pangenomes, particularly in plant genomes. It examines the structure of pangenome representation, including format comparison, conversion, visualization techniques, and their implications for enhancing crop improvement strategies.

**Key scientific concepts of review:**

Earlier comparative studies have illuminated novel gene sequences, copy number variations, and presence-absence variations across diverse crop species. The concept of a pan-genome, which captures multiple genetic variations from a broad spectrum of genotypes, offers a holistic perspective of a species’ genetic makeup. However, constructing a pan-genome for plants with larger genomes poses challenges, including managing vast genome sequence data and comprehending the genetic variations within the germplasm. To address these challenges, researchers have explored cost-effective alternatives to encapsulate species diversity in a single assembly known as a pangenome. This involves reducing the volume of genome sequences while focusing on genetic variations. With the growing prominence of the pan-genome concept in plant genomics, several software tools have emerged to facilitate pangenome construction.

This review sheds light on developing and utilizing software tools tailored for constructing pan-genomes in plants. It also discusses representation formats suitable for downstream analyses, offering valuable insights into the genetic landscape and evolutionary dynamics of plant species. In summary, this review underscores the significance of pan-genome construction and representation formats in resolving the genetic architecture of plants, particularly those with complex genomes. It provides a comprehensive overview of recent advancements, aiding in exploring and understanding plant genetic diversity.

## Highlights

• Recent NGS progress allows sequencing multiple genotypes per species, revolutionizing genomic analysis.
• Pan-genomes capture diverse genetic variations for comprehensive comparative analysis, especially in dioecious plants.
• Large plant genomes pose sequencing and computational challenges, addressed by methods like skim-sequencing and RNA-seq
• Emergence of specialized software tools aids in constructing pan-genomes, enhancing research efficiency in plant genomics.

## Graphical abstract

**Figure:**
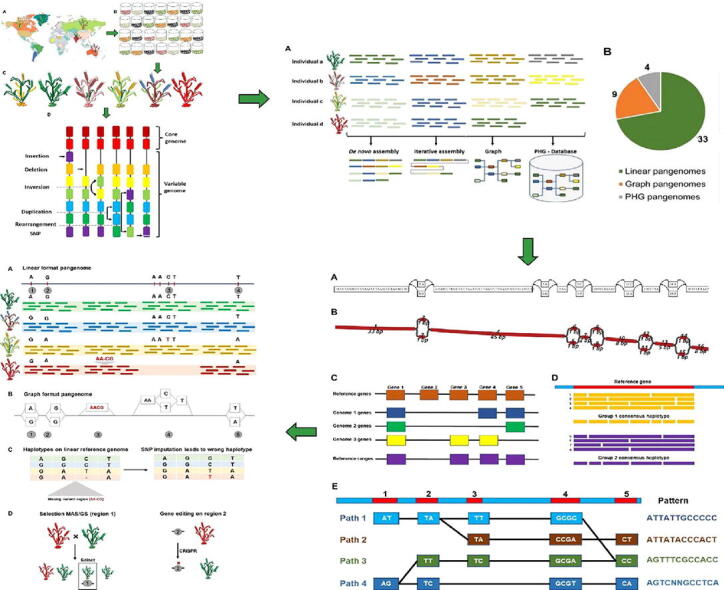

## Introduction

The single most important technology that has transformed genomics in the past decades is high-throughput DNA sequencing enabling cheap, fast and comprehensive generation of very large datasets on genomes. The availability of such huge volume of sequence data has been instrumental in the advancement of genomic studies for a wide range species including economically important crop.

Advanced genome sequencing systems incorporating Oxford Nanopore Technologies (ONT) and PacBio platforms alongside optical mapping and Hi-C technology have fundamentally transformed research in plant pangenomics. Further improvements in assembly technologies provide advanced tools for resolving complex plant genomes that bring together large amounts of repetitive DNA sequences and heterogenous structural variations that previous sequencing approaches could not handle. While initial efforts focused on generating high-quality reference genome assemblies for various plant species, such as maize [1], sorghum [2], soybean [3], potato [4], barley [5], chickpea [6], pigeonpea [7] and huge hexaploidy genome of bread wheat [8], [9]. Recent studies have revealed the limitations of relying solely on a single reference genome to capture the extensive genetic diversity present within a species [10]. The systems developed by ONT and PacBio constitute leading technologies that generate long-read sequence information. ONT provides live sequencing operations through its system to generate DNA reads measuring between several kilobases. The direct measurement of DNA molecules using this technology simplifies sample preparation requirements while expanding genomic analysis capabilities for both large and complex genomes. PacBio's premier long-read sequencing performance using SMRT (Single Molecule Real-Time) technology allows precise identification of ambiguous genomic regions including repetitive elements together with structural variants. Highly accurate reads from this technology are fundamental for reliable genome assembly and pangenome research outputs. These technologies have transformed plant pangenomic research through their ability to sequence complete genetic information from several different species members. The sequencing genomes provide information about both genetic diversity along with structural components and uncommon gene variants which help understand plant survival abilities. Our understanding of plant evolution together with environmental adaptation benefits from comprehensive genome variation detection. The integration of advanced sequencing technologies such as PacBio, HiFi, Oxford Nanopore, along with specialized assemblers like HiCanu [11], Falcon [12], and Hifiasm [13], has significantly impacted plant pangenomics research.

The technique of optical genome mapping alongside long-read sequencing serves as an essential tool for visualizing plant genome spatial arrangements. The technique creates genomic schematic maps with high-definition detail through visual observation of solitary DNA strands. This method maintains exceptional value when it functions to resolve gaps that exist in assembled genomes when sequencing data does not provide clear information. The Hi-C technology demonstrates extraordinary usefulness by giving scientists insights into chromosomal layer structures beyond previous knowledge. The Hi-C technique detects genomic loci spatial relationships to develop long-range genome interaction maps. Hi-C delivers significant value for identifying complex genomic regions containing repetitive elements along with large-scale structure variants. By capturing chromatin conformation Hi-C has delivered crucial information that helped reveal the functional characteristics of plant genomes alongside their pangenomic structures.Using these technologies, researchers have been able to dissect the complex genetic architectures of plant species containing brand-new genomic structures and evolved evolutionary processes driving fine-scale diversity in plants. In the last few years these new such sequencing technologies have been deployed in plant pangenome studies and played a major role driving discoveries with regards to important biological processes as well agricultural research providing tools for understanding both crop traits and mechanisams of adaptation.

The concept of a pan-genome, first introduced by [14], offers a complete representation of the entire genomic collection of a given species. A pan-genome is defined as the non-redundant collection of all DNA sequences present across all individuals within a species. This holistic approach to genomic representation has gained significant power in plant genomics, with pan-genomes being constructed for numerous crop species, including maize [15], soybean [16], rice [17], sorghum [18], [19], chickpea [20], [21], and wheat [22], [23].

Pan-genome analyses provide insights into the core genome, comprising the global genes across all individuals, and the dispensable or accessory genome, comprising genes specific to a subset of individuals. This distinction is crucial, as core genes are typically associated with essential biological functions and phenotypic traits, while accessory genes often contribute to specific adaptations and environmental responses [24]. By capturing this extensive genetic diversity, pan-genomes offer a powerful resource for understanding the evolutionary, functional, and phenotypic consequences of genomic variations within a species.

Moreover, pan-genomes have emerged as vital tools for crop improvement efforts, enabling the identification of genetic variants associated with desirable agronomic traits, such as yield, drought tolerance, and disease resistance. By leveraging the comprehensive genomic information provided by pan-genomes, researchers can focus on specific variants and develop molecular markers for marker-assisted selection or genomic prediction models, accelerating the breeding process and enhancing crop productivity.

This review provides a comprehensive overview of pan-genome construction approaches, data representation formats, and visualization tools, highlighting their applications in plant genomics and crop improvement. Additionally, it explores the potential of pan-genomes to revolutionize genomics-assisted breeding strategies, ultimately contributing to the development of improved crop varieties to address global food security challenges.

## Plant pan-genome analysis

**Fig. 1:**
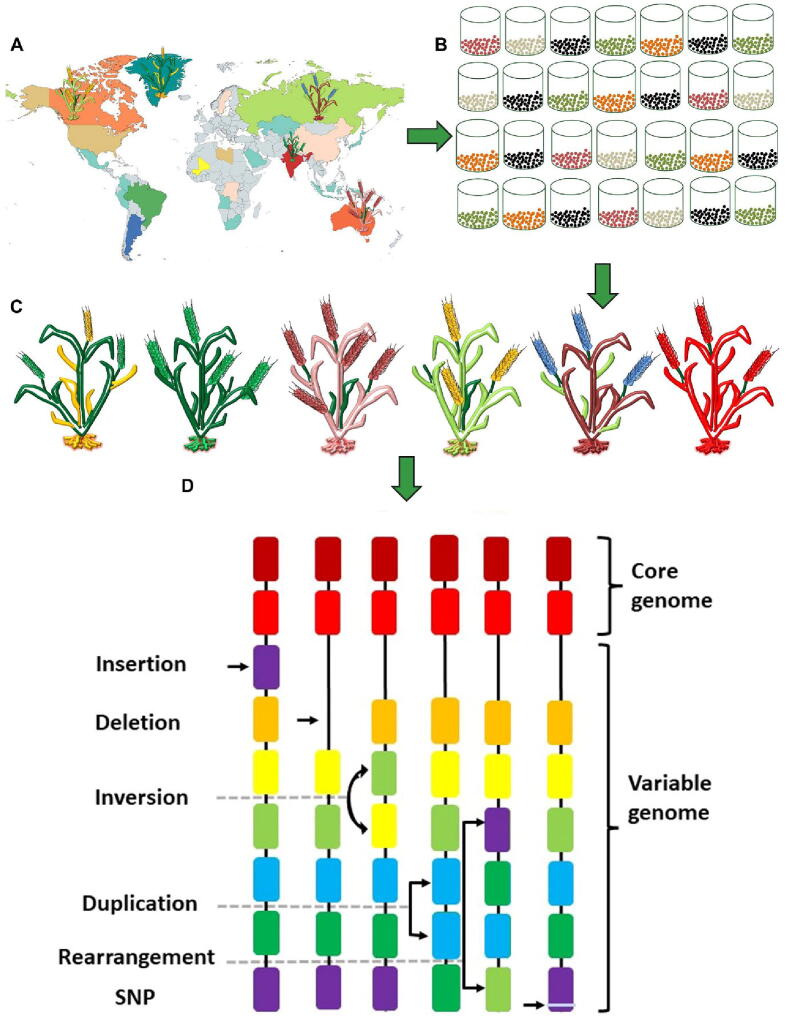
**Constructing pangenome from diverse global accessions:** A) Representative genotypes are chosen from genetically diverse global accessions; B) the accessions are preserved; C) cultivated to extract the genetic material, and; D) analysed to construct the pangenome through assembling the high-quality genomes and identifying the variations including both core and variable sequences of a species.

Next-generation sequencing technologies have transformed how researchers study genetic variations across crop species through developments in NGS technology (Fig. 1). The ongoing development of sequencing methods has resolved previous challenges with short read lengths and high error rates and non-uniform data coverage to facilitate precise genome investigation [25]. The pan-genome data structure enables storage of crop species or population genomic sequences which function as a reference framework to describe genomic collections across the pan-genome. Pan-genome models let researchers study complete species-wide genetic diversity through their ability to detect genomic variations that exist between individual specimens.

Constructing a pan-genome necessitates the availability of a complete set of haplotype-resolved genetic variations. Significant progress has been made in this regard through various HapMap projects, which aim to capture linkage information for species such as cassava [26], maize [27], rice (https://www.ncgr.ac.cn/RiceHap3/), and *Cajanus*spp. [28]. However, despite these advancements, sequencing reads often lack sufficient length to assemble all repeat structures, necessitating the integration of complementary technologies like array comparative genomic hybridization (aCGH), synthetic long reads, and high-throughput optical genome mapping to detect larger-scale structural variations (SVs) [29], [30].

Advanced sequencing methods present opportunities to integrate additional dimensional data including transcriptome profiling and DNA-protein interactions with epigenetic data for pan-genome investigations. Today's resequencing studies use whole-genome sequencing technologies to dive into genomic variations among different genotypes including single nucleotide polymorphisms (SNPs), insertions/deletions (indels) and large chromosomal structural variations. A comprehensive understanding of these genetic and genomic variants helps discover hazardous mutations and reveal plant domestication patterns and agricultural enhancement approaches [31].

The characterization of species becomes possible through pan-genome analyses which partition natural populations into core genome elements found across all individuals and overall pan-genome dimensions that include all genes or gene families represented within each population. Genomic and gene family analyses function at various levels according to the research design [32]. Essential biological processes along with major phenotypic traits constitute the core genome which exists alongside the accessory genome that includes genes able to adapt to environmental conditions and influence trait variability [33].

The research shows that core genes represent significant gene proportions across plant species where wheat contains 64 % and rice contains 89 % of total genes. When more genomes are studied variable genes linked to environmental adaptations become more prevalent throughout the core genome components.

Research on Brassica napus and wheat alongside sorghum and chickpea along with soybean has demonstrated that the pan-genome grows with additional genome sequencing, thus confirming open pan-genome organization is the dominant structural pattern in living plant species. The detection of additional genetic elements highlights the need for pan-genome methodologies which effectively capture a species' maximal genetic diversity so researchers can study phenotype-genotype interactions more thoroughly and accelerate crop enhancement programs.

## How is a pan-genome assembled?

**Fig. 2:**
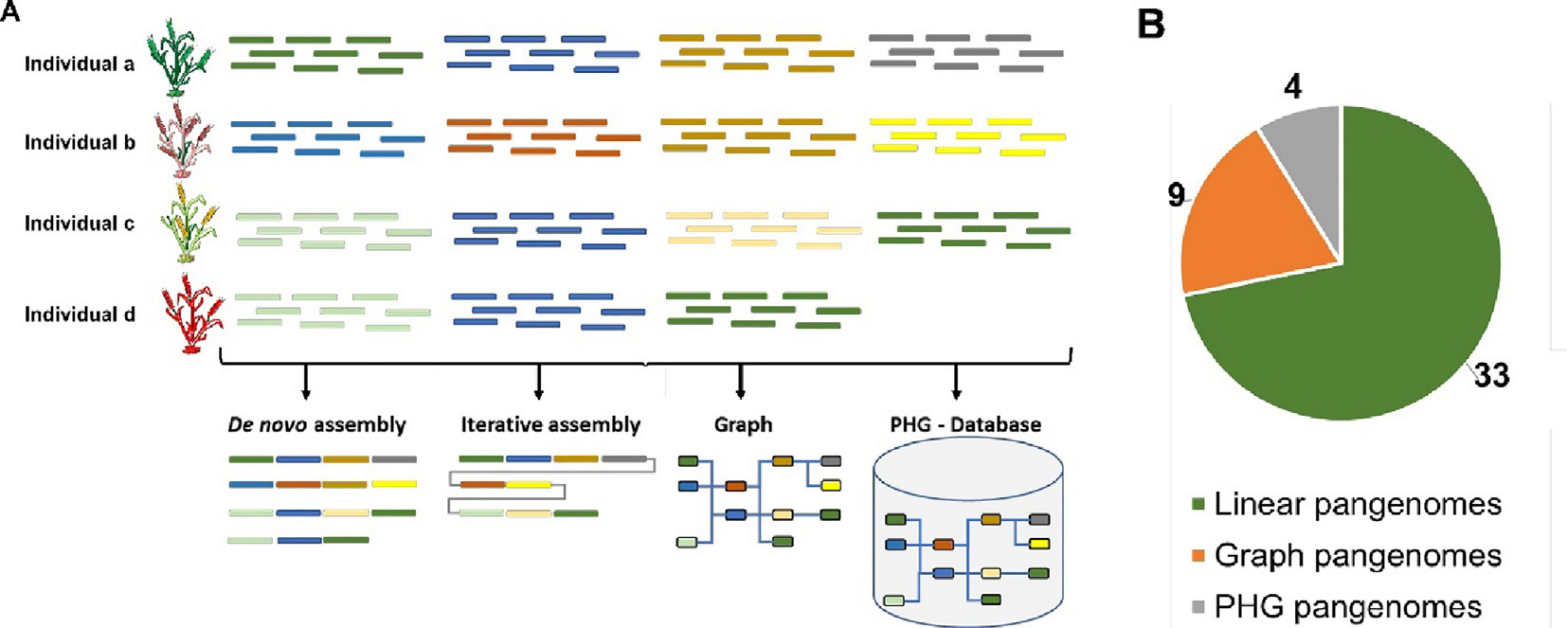
**Pangenome assembly methods and format:** A) Schema showing pangenome assembly methods. Sequence reads from individual genomes assembled using the *de novo* method (each color indicates gene for each individual) and compared to define the core and variable regions. In the iterative assembly, one of the genomes de novo was assembled and used as a reference for assembling the remaining genomes' unique sequences. Graph pangenome assembly represents the genes/sequences as interconnected nodes and each path represents the genome. In the PHG (practical haplotype graph), the nodes represent the reference ranges sequences and the graph stored in the database. B) The number of plant’ pangenomes assembled in each format. The linear format constitutes the major proportion of overall pangenome assemblies, followed by the graph and PHG format of assemblies.

The assembly of a pan-genome can be approached through either supervised or unsupervised (*de novo*) methods, depending on the availability of a reference genome. In the supervised approach, sequence reads from each cultivar are mapped to the reference genome, and only the unmapped reads are iteratively assembled. Conversely, the unsupervised approach does not require a reference genome, and the assembly process proceeds entirely *de novo* (Fig. 2) [32], [34].

Several bioinformatics tools have been developed to facilitate pan-genome construction from sequence datasets or assembled genomes. PANSEQ [35] and PGAP [36] enable the identification of novel genomic regions and the determination of additional features, such as single nucleotide polymorphisms (SNPs), core genome sequences, and accessory genome sequences. PANTOOLS [37], designed to handle large and complex genomes, can detect and annotate homologous regions within pan-genomic data.

In addition to collecting unique sequences, pan-genome assembly tools also consider minor variants between individuals within a crop species. These variants, ranging from SNPs and indels to larger structural variations, are represented as “bubbles” within a graph structure ordered by reference genome coordinates. Tools like GENOMEMAPPER [38], PANVC [39], GRAMTOOLS [40], GraphGenome Pipeline [41], PGGB [42], cactus [43], and VG [44] employ graph-based approaches to build such pan-genome representations.

The concept of the small variant graph extends further to genome assembly-level graphs. *De Bruijn* graph-based assemblers, such as Cortex [45], SplitMEM [46], TwoPaCo [47], and Bifrost [48], can adapt to pan-genome construction by assigning colours (representing specific biosamples) to nodes or unitigs. These coloured *de Bruijn* graphs enable population-scale analyses and facilitate the identification of sample-specific variations.

Furthermore, graph-based pan-genome data structures can be indexed to support efficient random access to elements and features within the graph, enhancing the utility of these resources for downstream analyses [49].

It is important to note that pan-genome assembly approaches can also incorporate transcriptomic data, providing an additional layer of information complementary to whole-genome sequences. Pan-transcriptome analyses capture partial genome information by cataloging the gene-level sequences present in each individual of a species. This approach enables the exploration of presence/absence variations (PAVs) and gene expression patterns, which can be integrated with genome-based pan-genome assemblies to enhance the resolution and accuracy of genetic variation detection.

Overall, the assembly of pan-genomes involves a diverse array of computational tools and methodologies, tailored to accommodate the inherent complexities of genomic data and the specific requirements of the target species or population under investigation. These approaches collectively aim to comprehensively capture and represent the genomic diversity within a species, laying the foundation for more advanced analyses and applications in crop improvement and breeding programs.

## How is a pan-genome represented?

The representation of a pan-genome can take various forms, each with its own strengths and limitations. The three primary formats for pan-genome data are: classical linear draft sequence format, assembly graphs, and practical haplotype graph database (PHG). Traditionally, the pangenomes are stored in the linear structure as collections of sequences in FASTA format. The variants in this linear format are stored in VCF format (small/structural variants). Whereas for graphical format pangenomes are stored in Graphical Fragment Assembly format, (GFAv1) (https://github.com/GFA-spec/GFA-spec/blob/master/GFA1.md) or Graph Alignment Format (GAF) (https://github.com/lh3/gfatools/blob/master/doc/rGFA.md#the-graph-alignment-format-gaf). The genomic features on the pangenome assembly are represented in a linear format (expecting the common co-ordinate system between physical and genetic position) compared to the other two forms (graph and PHG) (Fig. 2B). The read alignments on the graph pangenomes are stored in GAM format [44] and supported by tools such as VG, and GraphAligner [50].

### Linear sequence format pangenomes

A linear string of reference sequence base after the base in the pan-genome is a classical representation format, a standard FASTA format allowing visualization in two-dimensional genome browsers. In this representation, a novel sequence is identified from the individual genome sequence and either appended to the end of an existing reference sequence or inserted between prior known sequences. This format maintains the rearranged linear sequence bases with unique co-ordinate positions. The genetic variations between the cultivars, which could be as small as SNPs, insertions and deletions, or copy number variations, to as large as chromosomal rearrangements (deletions, duplications, inversions, and translocations), can report only one version of variation following the co-ordinate system (Fig. 1). To represent such a genome in a single linear format necessarily removes variations or finds an additional way of reporting such variations, and the co-ordinates system represents one of the genomic reference individual / parent. However, this format faces challenges in capturing the species-wide large population's specific features, like novel sequence, variations, similarity or functional content, so there is a need to address these challenges to represent species-wide information with proper genomic co-ordinate systems.

A FASTA format is a standard text-based format for representing nucleotide sequences in a linear format. The first line of each sequence starts with the ‘>’ symbol, followed by the sequence identifier (id), and the second line contains the actual series of sequence base characters. Many such pangenome assemblies have been developed recently for small genome crop *A. thaliana* to complex genomic structures like wheat (Table 1).

**Table 1:** The plant pangenomes published in linear format, graph and PHG format.

**Species**	**Domestication status**	**Ploidy**	**Number of accessions**	**Reference**
**Linear format**				
*Arabidopsis thaliana*	Crop	Diploid	69	[58]
*Brachypodium distachyon*	Wild	Diploid	54	[90]
*Brassica napus*	Crop	Tetraploid	53	[52]
*B. napus*	Crop	Tetraploid	50	[93]
*B. napus*	Crop	Tetraploid	9	[68]
*B. oleracea*	Crop	Diploid	10	[32]
Banana (*Musa and Ensete*)	Crop, hybrids	Triploid	15	[72]
*B*.*napus, rapa, oleracea*	Crop	Diploid, diploid, amphidiploid	87, 77 and 79	[97]
*B*.*rapa*	Crop	Diploid	3	[56]
Pepper (*Capsicum*)	Crop	Diploid	383	[62]
Chickpea (*Cicer arietinum*)	Crop	Diploid	3,366	[20]
Cowpea (*Vigna unguiculata*)	Crop	Diploid	6	https://doi.org/10.1101/2022.08.22.504811
Cowpea (Vigna unguiculata )	Crop	Diploid	6	[57]
Eggplant (*Solanum melongena*)	Crop	Diploid	23	[86]
*Soybean* (*Glycine soja*)	Wild	Tetraploid	7	[16]
Sunflower (*Helianthus annuus*)	Crop	Diploid	493	[51]
Walnut (*Juglan ssp.*)	Wild	Diploid	6	[77]
*Medicago truncatula*	Wild	Diploid	15	[88]
Melon (*Cucumis melo*)	Wild, landrace	Diploid	2	[63]
Mung bean (*Vigna radiata*)	Crop	Diploid	217	[54]
*Oryza sativa*	Crop	Diploid	3	[17]
*O. sativa* (indica/japonica)	Crop	Diploid	1,483	[30]
*O. sativa*	Crop	Diploid	3010	[85]
*O. sativa/ O. rufipogon*	Crop	Diploid	67	[79]
Pea (*Pisum sativum*)	Wild, Crop	Diploid	118	[84]
Pecan (*Carya illinoinensis*)	Tree	Diploid	4	[64]
Pigeon pea (*Cajanus cajan*)	Crop	Diploid	89	[80]
*Populus*	Tree	Diploid	19	[71]
*Populus*	Wild	Diploid	7	[74]
Potato (*Solanum tuberosum*)	Wild, Crop	Diploid	44	[67]
Sesame (*Sesamum indicum*)	Crop	Diploid	5	[82]
Tomato (*Solanum lycopersicum*)	Crop	Diploid	725	[91]
Sorghum (*Sorghum bicolor*)	Crop	Diploid	354	[19]
*Bread wheat* (*Triticum aestivum*)	Crop	Hexaploid	19	[22]
White lupin (*Lupinus albus*)	Crop	Diploid	39	[53]
Maize (*Zea mays*)	Crop	Tetraploid	503	[15]
Maize (*Zea mays* )	Crop	Diploid	721	[89]
**Graph format**				
*Arabidopsis thaliana*	Crop	Diploid	32	[61]
Broomcorn millet (*Panicum miliaceum*)	Wild	Diploid	32	[96]
Chickpea (*Cicer arietinum*)	Wild	Diploid	8	[21]
Cucumber (*Cucumis sativus*)	Crop	Diploid	11	[59]
*Soybean* (*G.* max)	Crop	Diploid	29	[55]
Grapevine *(Vitis vinifera)*	Wild	Diploid	9	[94]
Melon (*Cucumis melo*)	Crop	Diploid	3	[76]
Rice (*O. sativa*)	Crop	Diploid	33	[73]
Pepper (*Capsicum*)	Crop	Diploid	3	[62]
Radish (*Raphanus sativus*)	Crop	Diploid	11	[81]
Rice (*O. sativa*)	Crop	Diploid	251	[54]
Rice (*O. sativa*)	Crop	Diploid	12	[69]
Sorghum (*Sorghum bicolor*)	Crop/Wild	Diploid	16	[78]
Tea (*Camellia sinensis*)	Elit cultivars	Diploid	22	[95]
Tomato (*Solanum lycopersicon*)	Wild/Cultivated	Diploid	11	[60]
Tomato (*Solanum lycospersicum*)	Crop	Diploid	838	[87]
Grapevine (*Vitis vinifera* ssp. *Vinifera* )	Crop	Diploid	29	[66]
Brassica genomes	Crop	Tetraploid	41	[70]
Lattuce (*Lactuca sativa* )	Crop	Diploid	474	[83]
Barley (*Hordeum vulgare*)	Wild/Cultivated	Diploid	76	[92]
**PHG format**				
Cassava (*Manihot esculenta*)	Crop	Diploid	241	[65]
Maize (*Zea mays*)	Crop	Diploid	27	[75]
Sorghum (*Sorghum bicolor*)	Crop	Diploid	398	[18]
Wheat (*Triticum aestivum*)	Crop	Hexaploid	65	[98]

Eg:

>scaffold1

CGACGAACA>

NC_003071.7:19472573-19474387 *Arabidopsis thaliana* chromosome 2 sequence

TGATTTTCTAAAAGTAGAAGAAAATAAGTGCAGTCCATAAAATAAAATCCTATAAAAATGTTAAAACTAGATTCTTTTTTAAAAAACTAAAATTTGCTGCAGACATCTAAAATTTTCGAAAATGATTGGGTGGCTAAGA

### Graph format pangenomes

A graph-based pan-genome is an alternative format addressing the above issues with the linear format. The sequence graph serves to collapse the similar sequence into a single unique data structure that is still representative of the full set [99]. A *de Bruijn* graphs-based genome assembly is a popular graph representation in which each node represents a *k-mer,* and the edge represents an overlap of *k-1* bases between from and to nodes. A direct walk following the node labels can be interpreted as a DNA sequence [100]. A graph is bidirectional when it represents both strands of DNA and the inversions between them. A graph could also represent a pan-genome of multiple individuals capable of capturing all sequences and variations between individuals [99]. A genome graph representing the genome of a species (represents the whole genome relationship) will grow with genome information as more data on that species becomes available. Such a graph imposed with the linear co-ordinate system by constructing a linear ordering of the nodes can describe the pan-genome [101]. Based on the topological relationships between each individual graph, it is possible to construct a compressed graph format as implemented in a few software like splitMEM and VG, to construct bacterial and human pan-genome [69]. This is similar to the earlier demonstrated compact representation (splicing graph) for a collection of splicing variants [102] and its application was also adapted to transcriptome data [103], highlighting the importance of graph. The tools available to construct such representations (like cortex) and calling variants through assembly (like platypus and vg: Variant Graph) and newer ones are upcoming. A graph can be a solution for a single diploid genome, addressing the above-mentioned linear graph limitations. It can also be used to represent the genomes of multiple individuals, capturing all variation between them [104].

Additionally, a graph could be a coloured path representing a specific individual, where the path of the graph is annotated. Such a *de Brujin* graph has been implemented in software like cortex and platypus [45]. For graph representation, currently, three file formats (FASTG, GBZ, and GFA) have been developed and also implemented by a few assemblers, like ALLPATHS_LG and SPAdes, which produce fastg format, and ABYSS produces GFA format. A recently developed GBZ file format is a path-based format in which the sequences are the objects connected with the edges. It is a compressed format with a specialized C++ library developed for creating and reading the compressed graph file [105], although, the GBZ was not designed for assembly graphs. The available graph representation of a genome would allow mapping reads corresponding to variants available in the graph. The binary format graph, the ‘vg’ graph, has been developed to store sequence and variant information and inter-change the graph format [44].

#### FastG

FastG was the first format introduced (as FASTG) in 2012, which is an extension of the fasta format. The format mainly differed by representing edges as sequences, complicating the data operations (https://lh3.github.io/2014/07/19/a-proposal-of-the-grapical-fragment-assembly-format). A fastg format requires ‘begin’ and ‘end’ lines with each scaffold line starting with ‘>’ symbol. The below assembly example has two scaffolds named ‘scaffold1′ and ‘scaffold2′ in the fastg format.

Eg:

Fastg graph format

#FASTG:begin

#FASTG:version = 1.0:assembly_name=‘example’;

>scaffold1:scaffold1;

ACGANNNNN[5:gap:size=(5,4..6)]CATGGC

>scaffold2;

CGA[1:alt:allele|A,T]CGATCA

#FASTG:end;

Linear format (fasta)

>scaffold1

ACGANNNNNCATGGC

>scaffold2

CGACGATCA

#### Graphical format assembly (GFA)

Alternative to FastG, gfa is another format of a graph that is represented as a tab-delimited field like header (H), segment (S), link (L), containment (C) and path (P). GFA format was introduced in 2014, compatible with *de Bruijn* and *string* graphs. More specifications of this format are available at https://github.com/GFA-spec/GFA-spec, and the tools and API listed are available at the same link. Fig. 5. A and B below are the simple gfa format graph assembly with a string in reverse complement and a base mismatch.

Eg:

**Fastg graph format**

#FASTG:begin

#FASTG:version=1.0:assembly_name=‘example’;

>scaffold1:scaffold1;

CGACGA[1:alt:allele|A,T]CA

>scaffold2

ACGANNNNN[5:gap:size=(5,4..6)]CATGGC

#FASTG:end;

**Linear format (fasta)**

>scaffold1

CGACGA**A**CA

>scaffold2

ACGANNNNNCATGGC

### Practical haplotype graph (PHG)

Compared to the genome assembly graphs, the haplotype graph is a collection of nodes and edges for the sequence within the organism inherited from a single parent. The PHG is built from a subset of sequences (conserved sequences with genetic variations) called reference ranges. Such sequence ranges are represented as graph node, and the nodes are connected with edges, which do not contain the sequence range but indicate the two haplotypes were together in a particular individual [106]. The PHG represents the sequence of haplotypes instead of the complete nucleotide sequences and stores the data in the relational database format. For example, the existing genomic resources of the breeding program founder line (whole genome sequence data or whole genome assemblies) are loaded into a graph database. Such a database supports genomic analysis such as imputation of low sequence coverage (as low as 0.01x coverage) of individuals in the breeding population achieved based on consensus haplotypes derived from the graph database (https://bitbucket.org/bucklerlab/rphg/wiki/Home). The input sequence can be a whole genome sequence, a reduced representation sequence, or SNPs called from population data. The PHG database also stores an additional layer of genomic features with genic and intergenic haplotypes, assisting in annotating the haplotypes. The data is stored in the compact format of haplotypes in the form of an imputed path through the graph, resulting in a very compact storage of the graph path list of haplotypes for many genotypes in a relational database. Thus, the organized pangenome is finally formed by storing the node and edge relationship as the path for each individual.

**Fig. 3:**
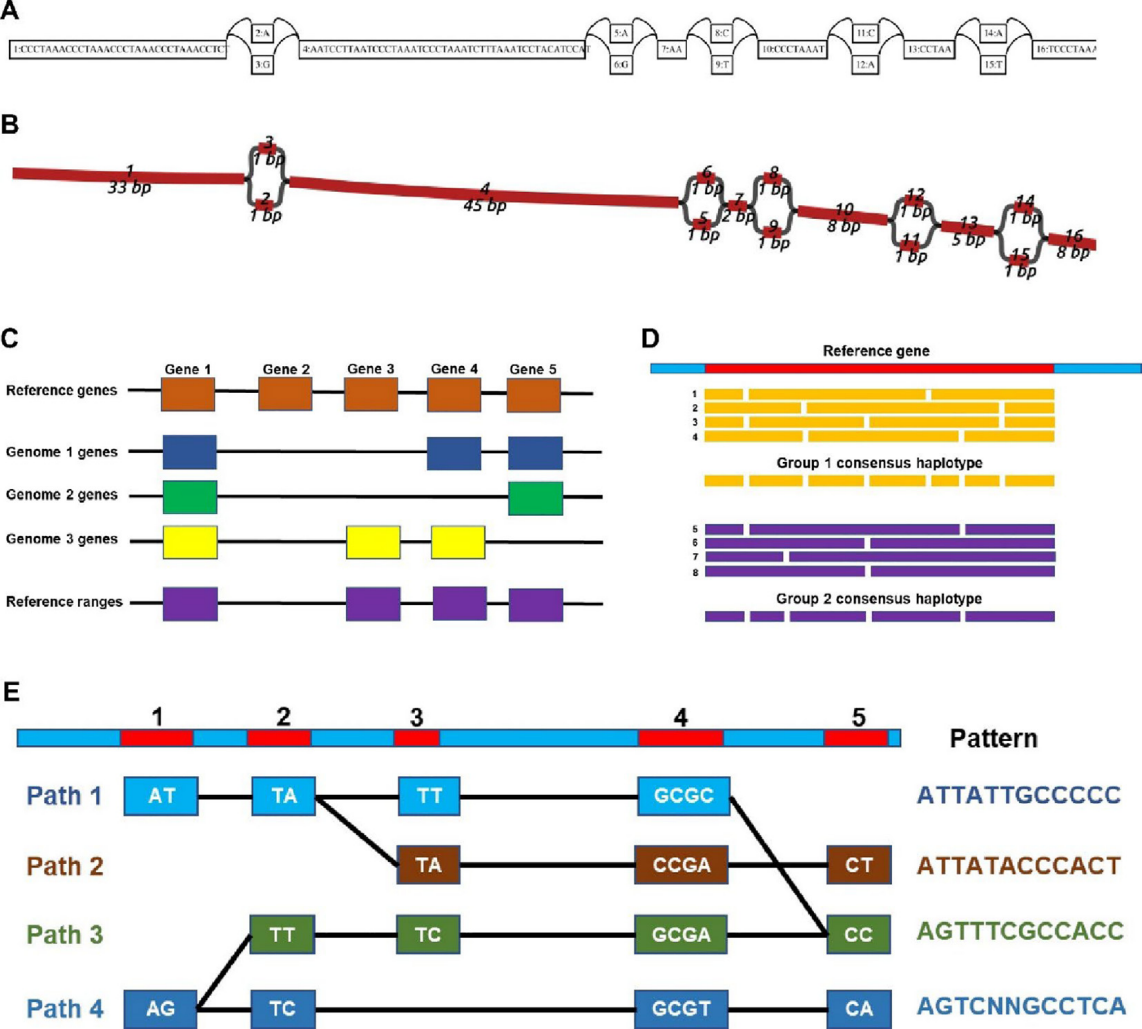
**A graph-based visualization in:** A) dot format viewer; B) graph format in Bandage The PHG database construction includes; C) identification of reference ranges/intervals sequences (conserved regions); D) identify the haplotypes and calling consensus for each group (of a population) and storing in the database; E) map the sequence read of a query individual of a population and follow the path to find the haplotypes from the database.

The first step of the PHG database is to assign the reference ranges in user-defined groups (e.g., gene and non-gene co-ordinates) followed by uploading to the database with haplotypes from other individuals [107] (Fig. 3). The database can be updated with either consensus haplotypes built from aligned genome assemblies or variants from WGS/reduced representation (GBS) data [107]. The PHG database has been implemented in sorghum [18], maize [75], wheat [98], and cassava [65] using the SNPs from diverse accessions WGS data and imputed with GBS/skim sequence data from inbred lines (Table 1). The PHG is deployed as a Docker image and available at https://hub.docker.com/r/maizegenetics/phg. Alternatively, a statistical programming language R package for PHG is available at https://bitbucket.org/bucklerlab/rphg/wiki/Home. HaploCart, working on the Bayesian inference principle is available in command-line and web interfaces [108].

## Formats comparison (linear vs graph)

The choice between linear and graph-based representations of pan-genomes has significant implications for capturing and analyzing genomic diversity within a species. Each format presents distinct advantages and limitations, shaping the types of analyses and applications that can be effectively performed.

**Fig. 4:**
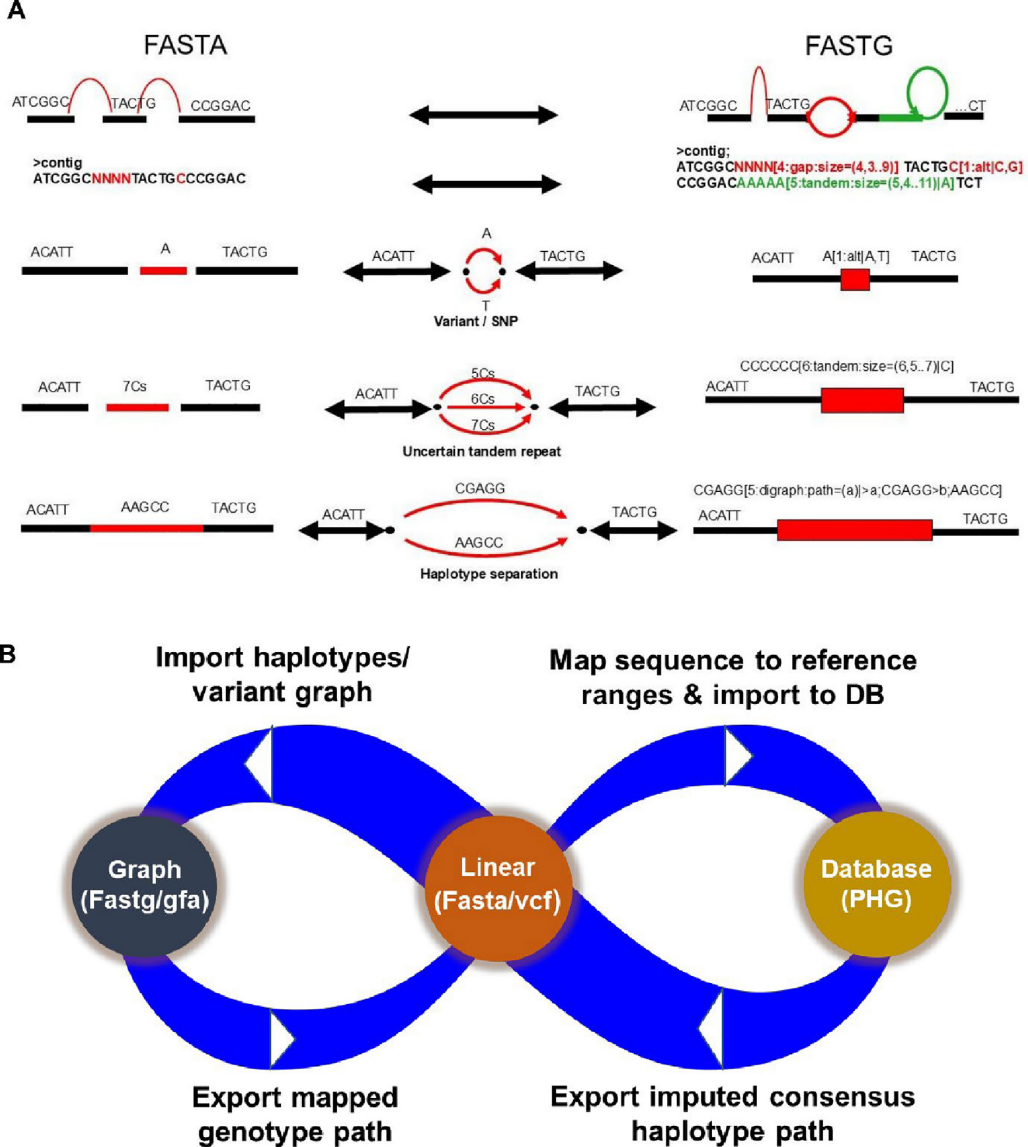
**Comparison of linear and graph formats in pangenome representation:** A) The linear and graph format sequence comparison. The linear format (FASTA) force to choose a random base (in case of SNP variant), a single path for uncertain repeat and haplotype patterns at a sequence position, whereas graph format (FASTG) encodes and store the genome complexity; B) A pangenome can be represented in a linear format, graph and PHG can interchange with few additional steps. A linear format can be converted to a graph with identified haplotypes/variants and export genotypes into a linear format. Similarly, a list of haplotypes called on reference ranges which are based on the linear format can be imported into PHG database in graph format and can export the imputed consensus haplotype path back to a linear format.

The linear sequence format, typically represented as FASTA files, has been the classical approach for representing genomic sequences. The first line of each sequence starts with the ‘>’ symbol, followed by the sequence identifier (id), and the second line contains the actual series of sequence base characters (Fig. 4). Many such pangenome assemblies have been developed recently for small genome crop *A. thaliana* (1001 genome project in Arabidopsis) to complex genomic structures like wheat [23] (Table 1).

Linear format offers several advantages, such as maintaining a straightforward coordinate system, enabling easy mapping of genomic features such as annotations, variants, and structural variations. The position of each base and the distance between bases are readily interpretable. Numerous bioinformatics tools and pipelines have been developed over decades to operate on linear sequence data, ensuring widespread compatibility and ease of integration with existing workflows (Compatibility). FASTA files are human-readable and require minimal computational resources, making them accessible and easy to manipulate.

However, linear representations also face significant limitations when it comes to capturing the full extent of genomic complexity within a species. The strictly linear nature of sequence representation forces assemblers to make arbitrary choices when encountering ambiguities, such as uncertain bases, single nucleotide polymorphisms (SNPs), or tandem repeats. This can lead to the loss of genetic information or the introduction of errors (Loss of information). Linear formats struggle to accurately represent structural variations, inversions, and complex haplotype relationships, as they can only accommodate a single representation of variations at a given coordinate (Inability to represent variations). As more genomes are added to a pan-genome, the linear representation becomes increasingly fragmented, reducing its utility and complicating downstream analyses (Limited scalability).

Graph-based representations, such as assembly graphs and variation graphs, offer an alternative approach that addresses many of the limitations of linear formats. Plant graph construction tools play a crucial role in capturing the complex genetic variations present in plant genomes, leading to the development of plant pangenomes. Recent advancements in graph construction tools have enabled researchers to construct comprehensive plant graph pangenomes, offering a more nuanced understanding of genetic diversity and evolutionary relationships within plant species.

Tools such as Cortex [45] and SplitMEM [46] have traditionally been used for graph construction in genomic studies, but their applicability in plant genomics may be limited due to the unique complexities of plant genomes. However, newer tools and methodologies tailored for pangenomes, such as VG (Variation Graph Toolkit) [44], PGGB [42], ODGI [109], cactus [43], and GraphAligner [50], have emerged to address these challenges more effectively. The tools like PGGB, cactus, and Minigraph-Cactus are alignment based graph generating tools applied for vertebrate and human pangenome studies [110], [111].

Graph structures can faithfully represent the non-linear complexities of genomes, including ambiguities, repeats, inversions, and structural variations, without the need for arbitrary decisions or loss of information (Preservation of complexity). Graph representations can accommodate variations across multiple individuals within a population, enabling population-scale analyses and the identification of sample-specific variations (Population-scale analysis). As new genomes are added to the pan-genome, graph structures can dynamically incorporate and represent the additional variations, providing a scalable framework for capturing genomic diversity (Scalability).

However, graph-based representations also face challenges. Unlike linear sequences, graph representations often lack a straightforward coordinate system for mapping genomic features, complicating analyses and requiring the development of specialized tools and methodologies (lack of coordinate system). Graph structures can be computationally intensive to construct, manipulate, and analyze, particularly for large and complex genomes (computational complexity). Effectively visualizing and interpreting the intricate patterns and relationships within graph-based pan-genomes can be challenging, requiring the development of specialized visualization tools (visualization challenges) listed in the Table 2.

**Table 2:** Plant pangenome visualization tools for linear and graph format assemblies.

**Software**	**Available site**	**Reference**
**Linear format**		
ABrowse (genome browser)	https://www.abrowse.org/	[116]
BasePlayer	https://github.com/rkataine/BasePlayer	[142]
Biodalliance	https://github.com/dasmoth/dalliance	[128]
Ensembl genome browser	https://useast.ensembl.org/Homo_sapiens/Location/View?r = 17:63992802-64038237	[143]
GBrowse 2	https://github.com/GMOD/GBrowse	[117]
GeneViTo	https://athina.biol.uoa.gr/bioinformatics/GENEVITO/	[126]
GenomeMaps	https://github.com/opencb/genome-maps	[125]
Gosling	https://gosling.js.org/	[113]
HiGlass	https://github.com/higlass/higlass	[145]
IGB	https://bioviz.org/	[123]
IGV	https://github.com/igvteam/igv	[121]
IGV.js	https://github.com/igvteam/igv.js/	[144]
JBrowse 2	https://jbrowse.org/jb2	[129]
Kero-BROWSE	https://kero.hgc.jp/examples/CLCL/hg38/index.html	[114]
NCBI Genome Data Viewer	https://www.ncbi.nlm.nih.gov/genome/gdv/	[140]
Nucleome browser	https://vis.nucleome.org/v1/main.html	[135]
pyGenomeTracks	https://github.com/deeptools/pyGenomeTracks	[127]
Tablet	https://ics.hutton.ac.uk/tablet/	[131]
Trackplot (python)	https://github.com/ygidtu/trackplot	[137]
Trackster	https://galaxyproject.org/learn/visualization/	[132]
UCSC genome browser	https://genome.ucsc.edu/	[146]
UTGB	https://utgenome.org/	[141]
Zenbu	https://fantom.gsc.riken.jp/zenbu/	[112]
**Graph format**		
AbySS-Explorer	https://github.com/bcgsc/ABySS-explorer	[134]
Assembly Graph Browser	https://www.github.com/almiheenko/AGB	[124]
Bandage	https://github.com/rrwick/Bandage	[119]
GfaViz	https://github.com/ggonnella/gfaviz	[133]
Icarus	https://bioinf.spbau.ru/icarus	[130]
IGV	https://igv.org/	[121]
MoMI-G	https://github.com/MoMI-G/MoMI-G	[139]
Panache	github.com/SouthGreenPlatform/panache	[122]
PanGraphViewer	https://github.com/TF-Chan-Lab/panGraphViewer	[136]
PGGB	https://github.com/pangenome/pggb	[42]
Ray Cloud Browser	https://deNovoAssembler.sf.Net/	[120]
SGTK	https://github.com/olga24912/SGTK.	[118]
VAG	https://ricegenomichjx.xiaomy.net/VAG/sequenceextraction.php	[115]
viralFlye	https://github.com/Dmitry-Antipov/viralFlye	[138]

Future developments in plant graph pangenomes may involve incorporating diverse genomic and epigenomic data (integration of multi-omics data) into plant graph pangenomes can provide a more holistic view of plant genomes. Advancements in graph algorithms and tools tailored for plant genomics (development of efficient graph algorithms) can enhance the accuracy and scalability of plant graph pangenome construction. Utilizing plant graph pangenomes for marker-assisted breeding and trait mapping can accelerate genetic improvement efforts in crops (application in breeding and crop improvement).

As pan-genome analyses continue to evolve, the choice between linear and graph-based representations will depend on the specific research objectives, the complexity of the target species, and the desired balance between comprehensiveness, computational efficiency, and interpretability.

## Is it possible to toggle between the formats?

The linear sequence and graph-based representations of pan-genomes are not mutually exclusive, but rather complementary approaches that can be leveraged in a coordinated manner. As Iain MacCallum and David B. Jaffe (from Broad Institute of MIT and Harvard, Cambridge) indicated, while each format has its unique strengths and limitations , it is possible to transition between them, capitalizing on their respective advantages for different stages of analysis or specific applications (Fig. 4).

### From linear to graph

Genome assembly tools, such as cloudSPAdes [147], ALLPATHS-LG [148], Cuttlefish 2 [149], and Minigraph-Cactus [111], typically employ a graph-based approach during the initial assembly process. These tools build assembly graphs by identifying overlapping sequence reads and representing them as nodes and edges. Subsequently, the optimal path through the graph is selected to generate the final non-branching assembly in a linear contig sequence format.

As more genome sequences become available for a species, the linear representation can be extended to accommodate variations from additional individuals by introducing “bubbles” or branches within the graph structure. This process effectively transitions from a linear format to a graph-based representation, enabling the capture of population-level variations and structural complexities.

### From graph to linear

Conversely, graph-based pan-genome representations can be linearized by exporting specific paths or haplotypes as linear sequences. This approach is particularly useful for integrating graph-based pan-genomes with existing bioinformatics pipelines and tools that operate on linear sequences.

For instance, in the construction of the wheat graph pan-genome, the gfatools gfa2bed utility was employed to linearize the graph representation, allowing the integration of genomic features and annotations from the linear coordinate system (https://doi.org/10.5281/zenodo.6085239).

In the case of Practical Haplotype Graphs (PHGs), the graph database can be queried with aligned sequence reads (e.g., from whole-genome sequencing or reduced representation sequencing) to extract linear haplotype sequences corresponding to specific individuals or accessions.

### Hybrid approaches

In many cases, a hybrid approach that leverages the strengths of both linear and graph-based formats may be advantageous. Linear representations can serve as a familiar coordinate system for mapping genomic features, annotations, and small-scale variations, while graph structures can capture the broader genomic diversity, including structural variations, inversions, and complex haplotype relationships.

This hybrid approach allows researchers to seamlessly transition between formats, utilizing linear sequences for downstream analyses and feature mapping, while leveraging graph structures for comprehensive representation of pan-genomic diversity and population-scale analyses.

Ongoing bioinformatics developments create tools that enable researchers to switch between linear and graph-based analytical formats through data structures which bridge the two methods. Pan-genomic data analysis becomes more comprehensive through ongoing tool improvements which allow researchers to leverage both formats' therapeutic possibilities [150]. Effective format interoperability techniques will unlock the complete potential of pan-genome analyses to push forward crop breeding research and agricultural development through paramount insights into species genetics.

## Visualization

Effective visualization is pivotal for interpreting and understanding the intricate relationships and patterns within pan-genomic data. While most visualization tools initially focused on linear reference genome structures, the increasing adoption of graph-based representations has necessitated the development of novel visualization approaches to capture the complexities inherent in these non-linear data structures (Table 2).

### Linear genome visualizers: Adapting to pan-genomic representations

**Fig. 5:**
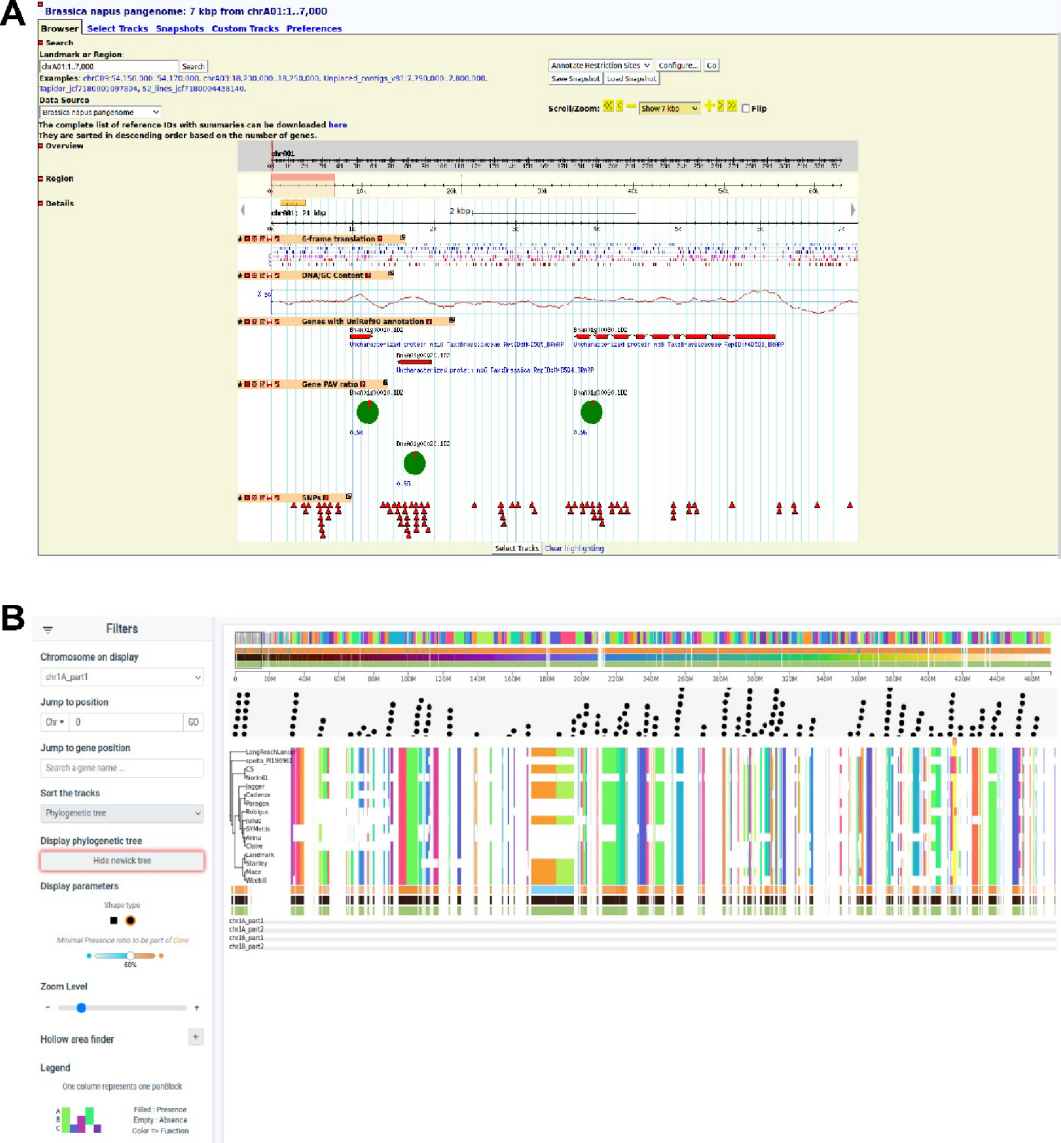
**Pangenome visualization of** A) a linear format pangenome in Gbrowse (*Brassica napus* pangenome); B) Panache screenshot of wheat pangenome.

Traditional linear genome visualizers, such as GBrowse, JBrowse2, and Circos, have been adapted to accommodate linear pan-genome representations (Fig. 5). These tools have been employed in various studies, including the visualization of pan-genomes for species like *Brassica napus*, *Brassica oleracea*, and wheat. While effective for linear sequences, these tools may struggle to accurately represent the intricate details and complexities present in graph-based pan-genomes.

### Graph-based genome visualization: Capturing non-linear complexities

To address the challenges of visualizing graph-based pan-genomes, several specialized tools have been developed for assembly graphs and variant graphs. Bandage is a visualization tool designed specifically for assembly graphs, capable of displaying connections and patterns within the graph structure. ODGI (Open, Decentralized Genomic Research) is a command-line tool for visualizing and analyzing assembly graphs. The tool for visualizing and exploring genome assembly graphs in the Graphical Fragment Assembly (GFA) format is possible with GfaViz.

Additionally, tools like vg view (part of the Variant Graph tool suite) and the Sequence Tube Map have been developed to visualize variation graphs at different scales, ranging from individual variations to larger structural variations. More tools are listed in Table 2.

### Network analysis and heatmap visualizations

Network analysis packages, such as igraph (available in Python, R, and C/C++), provide tools for visualizing and analyzing graph-based pan-genome data structures.

Moreover, heatmap visualizations have emerged as a powerful technique for representing shared genomic regions among individuals within a species. Tools like Panache can generate interactive web-based heatmaps, highlighting regions of similarity and divergence across different accessions or individuals.

### Visualizing practical haplotype graphs (PHGs)

For representations like Practical Haplotype Graphs (PHGs), which focus on capturing haplotype variations within a pan-genome, specialized visualization approaches are required. While tools exist for visualizing the linear components of PHGs (e.g., conserved sequence ranges), visualizing the haplotype connectivity and relationships within the graph structure remains an active area of development.

### Integrating multiple visualization approaches

As pan-genome analyses continue to advance, the development of effective visualization tools will be crucial for interpreting the intricate patterns of genomic diversity within species. By integrating multiple visualization approaches, ranging from linear genome browsers to graph-based representations and heatmaps, researchers will gain a comprehensive view of the genetic landscape, enabling deeper insights into the evolution, adaptation, and functional implications of genomic variations.

## Pangenomes towards the crop improvement

The introduction of pangenomic approaches may transform crop improvement by revolutionizing how we understand and utilize genetic diversity within species. Unlike traditional methods that depend on a single reference genome, pangenomics captures the full range of genetic variations, including both core genes found in all individuals and accessory genes present in only some. This comprehensive view of a species' gene pool provides new opportunities for identifying genetic variants linked to valuable agronomic traits, making crop improvement more precise and effective.

### Pangenomics for trait discovery

**Fig. 6:**
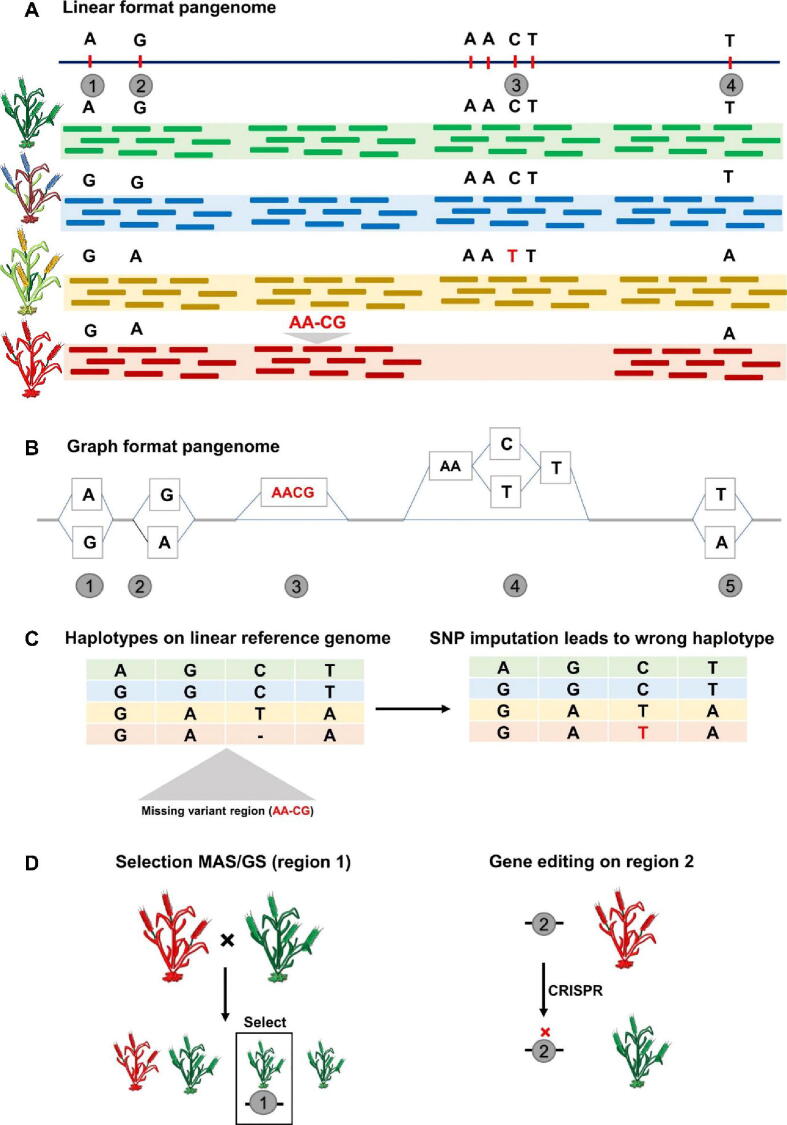
**The comparison of genetic variants in pangenome formats**: A) The variation was ignored between the 2nd and 3rd variation; B) whereas in graph format the same missing variation was captured; C) at the downstream analysis the haplotypes were missing leading to a wrong pattern of haplotypes and; D) With more accurate genetic information, breeders can utilize it to identify variants involved in MAS/GS and make alterations to the genome through genome editing.

Pangenomics represents a powerful crop improvement method because it enables researchers to study the actual genetic variants which determine traits including yield productivity mixed with drought resilience and disease immunity. Pan-genome-wide single nucleotide polymorphisms (SNPs) along with presence/absence variations (PAVs) provide high-density molecular markers which enable researchers to run powerful genome-wide association studies (GWAS) and quantitative trait locus (QTL) mapping analyses (Fig. 6). Through these methods scientists can discover quantitative trait nucleotides (QTNs) that correspond with desired phenotypic features to develop useful markers and genomic prediction models [66].

### Overcoming biases and capturing comprehensive genetic diversity

Pangenomics addresses the biases and limitations of using just one reference genome for genetic studies. It allows scientists to capture and study genetic variants that might be missing or underrepresented in a single reference genome. This leads to a more accurate and complete identification of genes linked to important traits. Additionally, pangenomics helps explore large genetic changes, such as copy number variations (CNVs) and gene presence/absence variations (PAVs), which significantly affect traits like leaf growth and disease resistance in crops like maize.

### Unleashing the potential of evolutionary dynamics and functional implications

Pangenomics allows scientists to study the differences in gene content across various plant types, including wild relatives. This helps them understand how these differences evolved and their impact on plant functions. By pinpointing gene families or specific genes linked to beneficial traits, researchers can use this information to introduce or edit these genes, speeding up the creation of better crop varieties with improved performance and desirable characteristics.

### Integrating pangenomics with advanced statistical models and machine learning

Combining pangenomic data with advanced statistical models and machine learning can greatly improve the accuracy of predicting genetic traits. By including structural variations, CNVs, and PAVs along with traditional SNP data in their models, researchers can get a fuller picture of the genetic makeup behind complex traits. This makes it easier to select and breed crops more accurately and efficiently, leading to better crop improvement programs.

### Fueling future advancements in crop improvement

The advancement of sequencing technology and wider availability of pangenomic crop species resources will allow more extensive utilization of pangenomic information for crop improvement programs. Pangenomic data integration alongside modern techniques including genome editing combined with genomic selection and gene introgression enables scientists to develop climate-adapted crop varieties exceeding the current yield limits and containing essential nutritional components that serve food security and environmentally-friendly farming systems [31], [151].

The future of modern agriculture depends heavily on pangenomic intervention to address genetic diversity needs and identify traits and create breeding precision methods while investigating genome-environment relationships and discovering new genetic material [150]. The wide-ranging genetic composition of crop species becomes accessible through pangenomics so it reshapes plant breeding approaches while fostering sustainable crop development suitable for evolving global agricultural requirements.

## Conclusion and future perspectives

Recent technological advancements have revolutionized the field of pangenomics, enabling the comprehensive representation of genetic variation within species through pangenome assemblies. These innovative approaches encompass both linear and graphical models, supporting sophisticated algorithms for sequence read mapping, visualization, and association studies. While graph-based pangenomes exhibit the capability to effectively relate multiple sequences, the debate persists regarding whether they will supplant the traditional linear reference genomes. Linear references offer the advantage of maintaining coordinate systems, enhancing their utility across diverse applications.

Identifying dispensable genes or sequences throughout a species' complete germplasm is a vital component of pan-genome research. However, the field faces notable challenges stemming from the limitations of existing technologies and computational programs. These limitations encompass issues such as the accuracy of gene annotations, the complexity of analyzing large-scale genomic data, the computational resources required for comprehensive pan-genome studies, and the need for standardized methodologies to ensure reproducibility and comparability across different studies. Overcoming these hurdles necessitates a detailed examination and refinement of current approaches to enhance the robustness and reliability of dispensable gene identification in pan-genome research.

Pangenome analysis offers valuable insights and tools that can significantly enhance crop breeding by facilitating the recovery of favorable genes lost in elite lines and integrating genome editing to guide future breeding strategies. Through pan-genome analysis, breeders can identify dispensable genes or sequences that are not present in all accessions but may confer beneficial traits under specific conditions. By investigating the functional roles of these dispensable genes, breeders can strategically reintroduce them into elite lines to enhance agronomic performance and resilience. The comprehensive understanding of dispensable genes provided by pan-genome analysis guides breeders in selecting and introgressing valuable genetic variants for trait improvement.

The genome editing method CRISPR-Cas9 creates precise and focused modification techniques to manipulate specific genes located in crop genomes. The utilization of pan-genome information helps breeders choose target genes for desired traits which they can modify using genome editing tools for novel alleles and deleterious mutation correction and gene expression level optimization in elite genetic lines. Through the use of this approach researchers can develop improved crop varieties with tailored benefits by accelerating breeding production and development cycles [151].

A critical challenge in pangenomics lies in addressing heterozygosity issues, where the presence of alternative alleles complicates variant identification. Strategies must be developed to differentiate true single nucleotide polymorphisms (SNPs) from variants arising exclusively due to heterozygosity during pangenome construction. Furthermore, there is a growing interest in generating taxonomically stratified pangenomes to elucidate variable genomic regions distinguishing taxa at species or family levels. Concurrently, conserved genomic regions hold promise for marker development to classify species taxonomically. Looking ahead, the prospect of creating pangenomes at higher taxonomic levels, such as the genus or family, or even a unified pangenome for viridiplantae, emerges as a fascinating avenue for future research endeavors with profound implications for evolutionary studies and biodiversity conservation efforts.

Machine learning and artificial intelligence (ML/AI) promise to enhance formatting and haplotype graphing operation (HG) within plant pangenomes through forthcoming studies that show predictive capabilities for genomic analysis techniques. Genomic research benefits increasingly from ML/AI technologies which create streamlined data analysis solutions for enhanced annotation accuracy alongside genome assembly results. In the realm of pangenome analysis, ML/AI algorithms can be leveraged to enhance the formatting of complex genomic data and improve the construction of haplotype graphs. By developing ML models that can recognize patterns in genomic sequences and structural variations, researchers can optimize the representation of pangenome graphs and accurately capture genetic diversity within plant species.

Moreover, the application of ML/AI in pan-genomic research extends to transcriptome assembly and annotation. By incorporating ML algorithms trained on small RNAs/microRNAs data, researchers can improve the efficiency and accuracy of pan-transcriptome assembly and annotation processes.

Future genomic endeavors will benefit from adding ML/AI methodologies to pangenome and pan-transcriptome analysis which provides enhanced accurate annotations combined with more advanced comparative genomics capabilities to reveal functional plant population variation.Embracing these technologies in future research endeavors can pave the way for innovative discoveries and transformative insights into plant genomic diversity and evolution.

## CRediT authorship contribution statement

**Pradeep Ruperao:** Conceptualization, Supervision, Writing – original draft, Visualization, Writing – review & editing. **Parimalan Rangan:** Writing – review & editing; Trushar Shah: Writing – review & editing. **Vinay Sharma:** Writing–original draft, Visualization, Writing–review & editing. **Abhishek Rathore:** Writing – review & editing. **Sean Mayes:** Writing – review & editing. **Manish K. Pandey:** Conceptualization, Supervision, Funding acquisition, Writing – original draft, Writing – review & editing.

## Declaration of competing interest

The authors declare that they have no known competing financial interests or personal relationships that could have appeared to influence the work reported in this paper.
